# Molecular Dynamics of Poly(Ethylene Glycol) Intercalated in Clay, Studied Using ^13^C Solid-State NMR

**DOI:** 10.3390/ma6010047

**Published:** 2012-12-21

**Authors:** Roberto W. A. Franco, Carlos A. Brasil, Gerson L. Mantovani, Eduardo R. de Azevedo, Tito J. Bonagamba

**Affiliations:** São Carlos Institute of Physics, University of São Paulo, P.O.Box 369, São Carlos, SP 13560-970, Brazil; E-Mails: franco@uenf.br (R.W.A.F.); carlosbrasil@ifsc.usp.br (C.A.B.); gerson.mantovani@ufabc.edu.br (G.L.M.)

**Keywords:** molecular dynamics, polymer intercalation, layered silicates, smectite clays, polymer electrolytes, Exchange NMR, nanocomposites

## Abstract

In this study, Cross-Polarization Magic-angle Spinning CP/MAS, 2D Exchange, Centerband-Only Detection of Exchange (CODEX), and Separated-Local-Field (SLF) NMR experiments were used to study the molecular dynamics of poly(ethylene glycol) (PEG) inside Hectorite/PEG intercalation compounds in both single- and double-layer configurations. The results revealed that the overall amplitude of the motions of the PEG chain in the single-layer configuration is considerably smaller than that observed for the double-layer intercalation compound. This result indicates that the effect of having the polymer chain interacting with both clay platelets is to produce a substantial decrease in the motional amplitudes of those chains. The presence of these dynamically restricted segments might be explained by the presence of anchoring points between the clay platelets and the PEG oxygen atoms, which was induced by the Na^+^ cations. By comparing the PEG motional amplitudes of the double-layered nanocomposites composed of polymers with different molecular weights, a decrease in the motional amplitude for the smaller PEG chain was observed, which might also be understood using the presence of anchoring points.

## 1. Introduction

The intercalation of organic compounds into layered clay minerals has been widely studied during recent years, which has been inspired not only because of their important technological applications but also because they are appropriate systems for studying the fundamental aspects of polymers confined within nanostructures [1,2,3,4,5,6]. 

One of the most interesting applications of these nanocomposites involves the intercalation compounds composed of smectite clays and poly(ethylene oxide) (PEO) or poly(ethylene glycol) (PEG), which are both solid polymer electrolytes (SPE), due to their structural and dynamical properties, which are very useful for solid-state secondary batteries because of their ionic conducting properties [7,8,9]. Therefore, knowledge of the conformational and dynamical characteristics of PEO or PEG inside these nanocomposites is a requirement for understanding their properties. Despite the fact that PEO and PEG are essentially the same polymers (they have the same repeat units), they are distinguished in this paper because of their molecular weights, where PEG possesses the smaller molecular weights (≤10,000 g/mol). 

Solid-state NMR is one of the methods that can be used to study these materials, and it can supply important information about them. Recently, several studies on PEG or PEO intercalated in clays have been performed. The conformations of the OC–CO bonds of both neat PEG and PEG intercalated in hectorite have been investigated using ^13^C double-quantum solid-state NMR [10,11]. The results of these studies indicated that the OC–CO bond angles of the PEG chains confined in the intercalation gaps are ~90% in the *gauche* (~70°) and ~10% in the *trans* (~180°) conformations, which provides valuable constraints on the possible chain conformation in the intercalation gaps.

The dynamical behavior of the PEO chain and the mobility of the Li^+^ ion (present in the clays as a charge compensation cation) were thoroughly investigated by Zax* et al.* [12,13,14]. The authors were able to obtain structural and dynamical information using solid-state NMR methods, even for highly disordered solid materials that contained paramagnetic impurities such as hectorite. T_1_ studies of ^7^Li and ^2^H (used in place of ^1^H by isotopic enrichment along the PEG chains) suggested that the some of the dynamic processes are responsible for spin-lattice relaxations for both the polymer and the cation. The narrowing of the ^7^Li NMR lines of the cations located at the interlayer Li sites suggested that the cation dynamics could be probed independently of the polymer dynamics. The timescale for the cation jumps was observed to be substantially longer than the timescale for polymer reorientation at temperatures below 340 K. The cation environment in the vicinity of Li^+^ differs substantially from that observed in traditional SPEs, and this difference is expected to be correlated to the difference in the cation mobilities. Indeed, the natural separation of the cation from the bulky anion formed in this nanocomposite allowed the authors to analyze the cation dynamics independent of the mobilities of the polymer host and the counter anion. It was demonstrated that the diffusion of Li^+^ in the PEG/clay nanocomposites is a complex phenomenon. Unlike the situation commonly encountered in solid polymer electrolytes based on Li salts dissolved in PEG, the limitation on the diffusivity in these compounds would appear to be an inefficient coordination of the Li^+^ ion to the backbone oxides in the polyether. Consequently, the ion dynamics in these nanocomposites appear to involve sticky hops along the oxide layer of the silicate. Additionally [15], the authors improved their knowledge about the dynamical and structural properties of the PEG/hectorite intercalation compounds using ^2^H NMR, measuring 1D spectra* versus* temperature and 2D Exchange spectra. They observed two competing influences on the dynamical properties of polymers chains with molecular weights greater than 10,000 g/mol. 

In reference [16] it was observed using ^1^H and ^13^C NMR experiments that the PEO chains with an average molecular weight of 400,000 g/mol intercalated in montmorillonite exhibit large amplitude motions, even at temperatures below the glass transition of neat PEO. The spectral density of the motions was observed to be high in the range of 50 kHz across a wide temperature range, which included the glass transition temperature and the melting temperature of neat PEO. The data also suggested that the conformation and type of motion for neat and intercalated PEO are similar and consist of helical jumps; however, the jump motion in the nanocomposite exhibits a lower apparent activation energy. 

In reference [17] it was clearly shown using ^1^H–^29^Si Wideline Separation WISE with ^1^H spin-diffusion NMR experiments that there is a mobility gradient of intercalated PEG segments that have a molecular weight of 10,000 g/mol. The results indicated that there are segments that not only present restricted mobility close to the clay surface (~0.5 nm), as expected for intercalated PEG, but also other segments that present a higher mobility more distant from the platelets (>1 nm). In other words, some of the PEG segments would be intercalated, whereas part of them would partially or completely extrude from the interlayer space. 

From the theoretical point of view, atomistic computer modeling of PEO-montmorillonite nanocomposites [18,19,20,21,22] revealed that, due to both (i) the electrostatic attraction between the clay surface and the Li^+^ cations and (ii) the more efficient coordination of the Li^+^ cations with the oxygen atoms on the clay surface compared to the Li^+^ PEO coordination, the Li^+^ cations are located in close proximity to the surface of the clay. Because the Li^+^ cations are also coordinated to oxygen atoms from adjacent polymer chains and are also attracted to the surfaces of the clay, each individual cation acts as an effective “anchor” point, which binds polymer chains to the confining wall. The greater the number of charges present in the clay surface, the more PEO oxygen atoms participate in coordination, which leads to a greater extent of binding for the chains. 

In this study, we used Exchange [23,24,25] and Separated-Local-Field [26,27] NMR experiments to obtain additional understanding about the dynamical and conformational aspects of poly(ethylene glycol)/Na^+^-Hectorite intercalation compounds in both single- and double-layer configurations. Three different PEG molecular weights, 200, 2000, and 10,000 g/mol, were used to study the possible Na^+^ anchoring effects on the polymer dynamics. The primary objective of this work was to study the polymer motional amplitudes in intercalation PEG/Na^+^ Hectorite nanocomposites, compare different layer configurations and molecular weights to provide new experimental evidences of the presence of anchoring points between clays platelets and PEG oxygen atoms and to determine their effects on the polymer motional dynamics.

## 2. Results and Discussion

### 2.1. Sample Characterization

**Figure 1:**
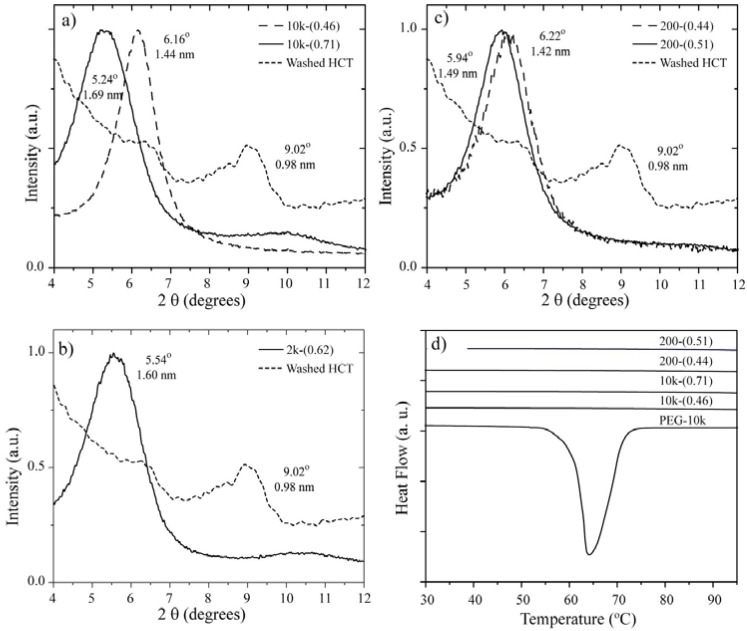
(**a**)–(**c**) WAXD curves for pure/washed HCT and the following nanocomposites: 10k-(0.46), 10k-(0.71), 2k-(0.62), 200-(0.44), and 200-(0.51); (**d**) DSC curves for pure PEG with M_w_ = 10,000 g/mol and the following nanocomposites: 10k-(0.46), 10k-(0.71), 200-(0.44), and 200-(0.51).

The WAXD data of the nanocomposites exhibited an average increase in the separation of the original inorganic galleries by *ca.* 0.45 or 0.67 nm (Figure 1a–c), which indicates that the PEG and the hosts formed nanocomposites with single and double PEG layers, respectively [7]. The long period of the inorganic layers, c, was obtained from the (001) peak. Table 1 summarizes the long period, c, of the inorganic layers before and after the intercalation of PEG, which was obtained from the WAXD experiments for each sample studied. The WAXD measurements also confirmed the suppression of the crystalline phase of PEG (not shown in the figure). 

Figure 1d presents the DSC curves recorded in the range of 30 to 100 °C for neat PEG (10,000 g/mol) and the intercalated nanocomposites. This figure shows the characteristic behavior of neat PEG, where the large endotherm at 67 °C corresponds to the melting of the crystalline regions. The absence of a melting transition for all of the nanocomposites indicates that the presence of the inorganic layers prevented the crystallization of the PEG [2,7,9,28,29,30]. 

**Table 1:** Composition and interlayer distances of the samples.

Sample	R	Host c_0_ (nm)	Nanocomposite c_1_ (nm)	Δc (nm)	Layer
10k-(0.46)	0.1	0.98	1.44	0.46	Single
10k-(0.71)	0.4	0.98	1.69	0.71	Double
2k-(0.62)	0.4	0.98	1.60	0.62	Double
200-(0.44)	0.1	0.98	1.42	0.44	Single *
200-(0.51)	0.4	0.98	1.49	0.51	Single *

* Independent of the amount of polymer used to prepare the nanocomposite using PEG with M_w_ = 200 g/mol, only monolayer materials were obtained. For the NMR experiments, we only used sample 200-(0.44).

### 2.2. ^13^C Line Shape *versus* Temperature

The ^13^C chemical shift spectra (or ^13^C CSA spectra) are very useful for identifying the presence of dynamical processes in polymers. This determination is relatively straightforward for PEG because it only contains one magnetically non-equivalent carbon along the polymer chain. We used the ^13^C CSA spectra to obtain a qualitative description of the molecular motion in the different samples. 

**Figure 2:**
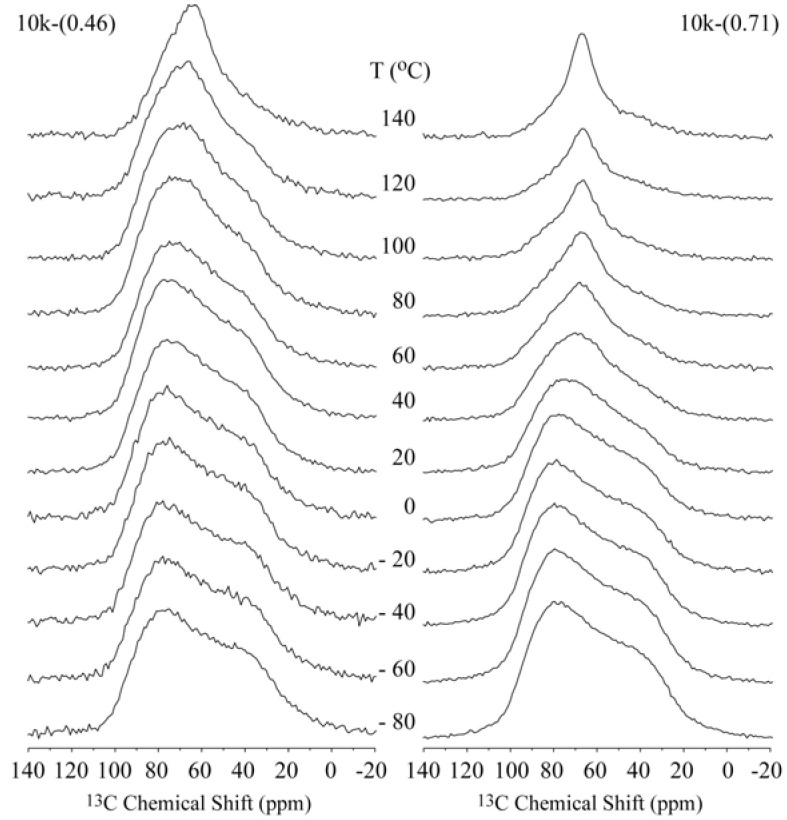
Temperature dependence of the ^13^C NMR spectra obtained for the single- and double-layer nanocomposites 10k-(0.46) and 10k-(0.71), respectively.

**Figure 3:**
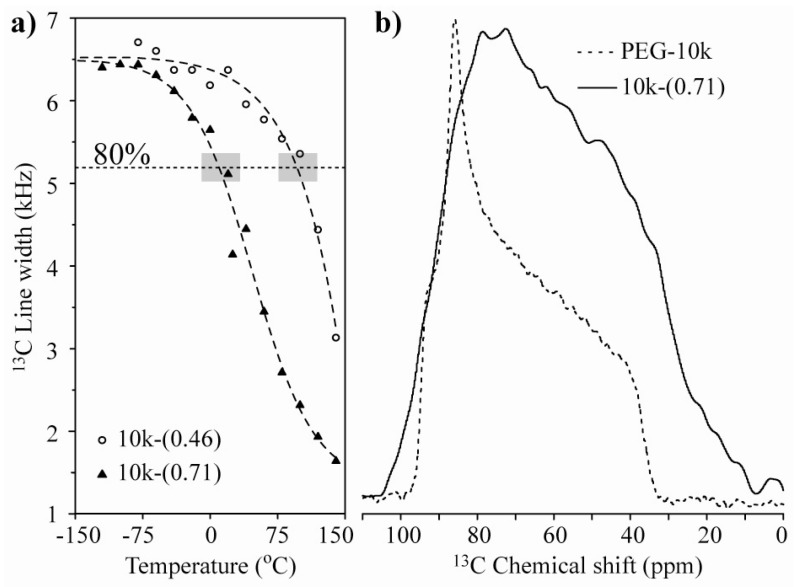
(**a**) ^13^C line-width* versus* temperature for the 10k-(0.46) and 10k-(0.71) samples. The horizontal dashed line and the gray boxes represent the regions where the line-widths correspond to approximately 80% of their rigid limit values; (**b**) Room temperature ^13^C NMR spectra for pure PEG with M_w_ = 10,000 g/mol and the 10k-(0.71) samples.

Figure 2 shows the temperature dependence of the ^13^C CSA spectra for the 10k-(0.46) and 10k-(0.71) samples, which correspond to the single- and double-layer PEG configurations, respectively. In both cases, the narrowing of the spectra is clearly observed, which occurs at lower temperatures for the 10k-(0.71) sample. Figure 3a presents the plots of the line width at half maximum* versus* temperature for the 10k-(0.46) and 10k-(0.71) nanocomposites. The observed behavior suggests that the chain motion in the single-layer intercalated compound has a higher activation energy, which might be related to a stronger interaction with the clay platelets. To obtain a better picture of the motion, we studied the motional geometry using Exchange NMR experiments. However, to compare the motional amplitudes in different samples, it is preferable for the motions to have similar correlation times (similar dynamic regimes). To achieve this behavior, the measurement temperature must be chosen in such a way that the motionally averaged powder spectra for both samples are similar, which can in fact be easily obtained by selecting the temperature where the line widths are as similar as possible. Therefore, all of the Exchange experiments presented in the following sections were performed at temperatures where the line widths at half maxima correspond to approximately 80% of their rigid limit values; see the gray boxes in Figure 3a. Note that the powder spectra observed for the lowest temperatures are broader than that observed for pure PEG (Figure 3b). This broadening is primarily due to magnetic susceptibility effects in HCT. For naturally occurring clays, the occurrence of magnetic impurities is well-known [31,32,33] If these impurities were in the paramagnetic phase, they would produce a strong reduction the in relaxation times that could be so severe that they would render NMR signals completely unobservable [34]. However, it was reported in reference [35] that the true magnetic susceptibility of the HCT matrix is very small, and the measured high low-field M/H ratio is actually due to the presence of small amounts of ferrimagnetic Fe_3_O_4_ and/or *γ*-Fe_2_O_3_ oxides, which exhibit ferromagnetic-like behavior that saturates at low fields (~5 kOe) and do not drastically influence the high-field (~94 kOe) NMR spectra and relaxation times.

### 2.3. 2D Separated-Local-Field NMR 

To obtain information about the geometry of the PEG molecular motion in the intercalated compounds, we used 1D and 2D Exchange NMR experiments. However, the interpretation of the results depends on the knowledge (or at least an estimation) of the orientation of the principal axis system (PAS) of the chemical shift tensor relative to a fixed local molecular axis frame (MF). To achieve this requirement, 2D Separated-Local-Field (SLF) experiments [26,27] were performed for pure PEG with a M_w_ = 10,000 g/mol and for the 10k-(0.71) samples. This experiment provides a 2D powder spectrum that corresponds to the correlation between the ^13^C–^1^H dipolar and ^13^C chemical-shift anisotropic (CSA) interactions. This correlation depends on the principal values and the PAS orientation of both the ^13^C–^1^H dipolar and ^13^C chemical-shift tensors. The CSA principal values were obtained from the ^13^C powder spectrum. The ^13^C–^1^H dipolar interaction principal values are easily obtained from the typical C–H distance, and the orientation of the main axis of the C–H dipolar interaction tensor is directly obtained from the orientation of the ^13^C–^1^H bond. Consequently, the only unknown variable that defines the 2D SLF spectrum is the PAS orientation of the chemical shift tensor. This PAS orientation is given by the three Euler angles (*α*, *β*, *γ*) that define the relative orientation between the C–H dipolar and CSA tensors. Thus, by simulating the 2D SLF spectrum for a set of Euler angles and comparing it with the experimental one, we can obtain these angles (*α*, *β*, and *γ*) using a best-fit analysis. For the CH_2_ groups, the same analysis can be performed, but in this case, the two CH dipolar tensors are replaced by the sum and difference tensors, which are also well-defined because the bond angle between the two CH bonds is fixed [27].

**Figure 4:**
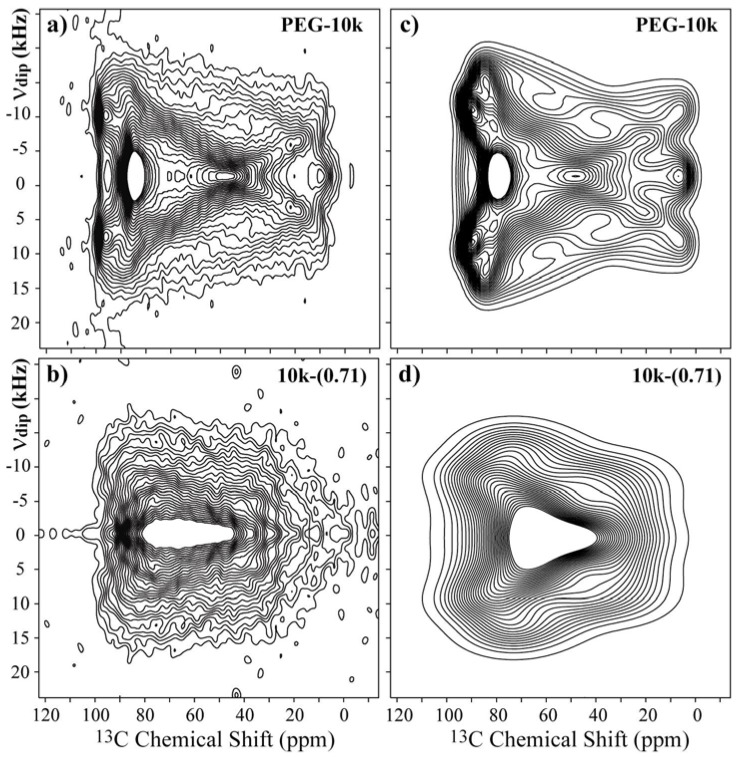
Experimental 2D Separated-Local-Field NMR spectra obtained for (**a**),(**b**) pure PEG with M_w_ = 10,000 g/mol and the 10k-(0.71) samples at room temperature; and (**c**),(**d**) the corresponding simulations using the CSA orientation reported in reference [27].

Figure 4a,c present the experimental and best-fit simulated SLF spectra for the pure PEG with a M_w_ = 10,000 g/mol. The experimental spectrum can be well reproduced using the set of Euler angles (90°, 20°, and 90°). For the 7_2_ helix conformation of PEG in the crystalline phase, this orientation means that the *σ_yy_* principal axis is along the middle line of the two CH bonds, and the *σ_zz_* principal axis lies 25° from the local helix axis. These values are in good agreement with those obtained in a previous study given the estimated imprecision of about 5° in the determination of the Euler angles [27]. The 2D SLF spectrum for the 10k-(0.71) sample is shown in Figure 4b. 

As observed, this spectrum is significantly different from that of pure PEG, but the symmetries of both spectra are similar. In fact, the majority of the differences between the spectra are attributed to the strong broadening of the powder spectrum. Thus, to simulate the SLF spectrum for PEG in the 10k-(0.71) sample, we took the principal values from the ^13^C powder pattern and assumed that the structure of PEG inside the clay is essentially a distorted helix [10], which allowed us to assume a local chain axis quite similar to that of pure PEG. Therefore, we simulated the spectrum using the orientations obtained from pure PEG as a base, and the best simulation was obtained for the angles (90°, 30°, and 90°), Figure 4d. However, note that despite having the correct symmetry, the reproduction of the experimental spectrum by the simulated one is not as good as for pure PEG. Indeed, due to the conformation disorder along the PEG helix in the intercalated compound, which produces a distribution of chemical shifts and a broadening due to susceptibility effects, the precise Euler orientations cannot be obtained from these results, which were used only as a hint of the basic Euler orientations used in 2D Exchange simulations. In other words, the CSA orientation (90°, 30°, and 90°) was used as a starting point for the 2D Exchange simulations, and the CSA distribution (amplitude and orientations) were included in the 2D Exchange simulations by using a proper reorientation angle distribution [36] and an artificial Gaussian broadening. Thus, in this work, we used the basic CSA orientation obtained from the 2D SFL experiments and introduced the disorder in the reorientation angle distribution.

### 2.4. Exchange NMR 

**Figure 5:**
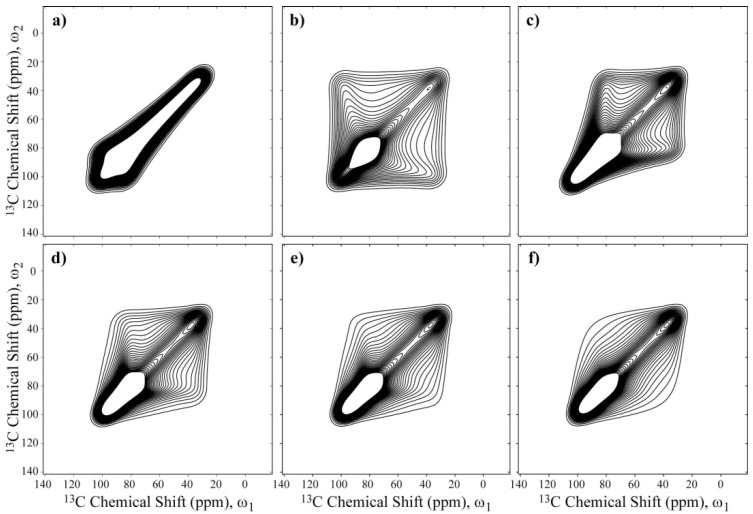
Simulations of 2D Exchange NMR spectra considering a Gaussian distribution of rotation angles with a root-mean-square angle of 60°. Rotations around the (**a**) *σ_xx_*; (**b**) *σ_yy_*; and (**c**) *σ_zz_* principal axes, and around axes (**d**) 30°; (**e**) 40°; and (**f**) 50° apart from the *σ_zz_* principal axis. In these cases, the angle *β* corresponds to the Euler angle that relates the *z*-axis of the local chain and the PAS of the chemical shift tensor.

2D Exchange NMR [23] is a powerful technique for studying slow molecular reorientations. This measurement essentially consists of monitoring the orientation-dependent NMR frequencies before (*t*_1_) and after (*t*_2_) a mixing time (*t*_m_), when usually *t*_1_*,t*_2_ ≪ *t*_m,_ such that the molecular reorientation only can occur during *t*_m_. In the absence of molecular motion, the 2D spectrum obtained after the 2D Fourier transform of the 2D time signal S(*t*_1_,*t*_2_,*t*_m_) in *t*_1_ and *t*_2_, is purely diagonal. In contrast, if slow molecular reorientations occur during *t_m_* , off-diagonal intensities are observed. The intensity pattern of the 2D Exchange spectrum strongly depends on motional geometry,* i.e.*, it depends on the motional amplitude and the rotation axis, which allows this parameter to be estimated from this experiment. This feature makes the 2D Exchange method particularly powerful in this work, where the primary objective is to determine the differences between the motional behaviors based on the composition of the intercalation compounds. Using the Euler angles for the PAS orientation of the chemical shift, we investigated the dependence of the rotational axis in the 2D Exchange Experiment by conducting a set of simulations considering a Gaussian distribution of rotation angles with root-mean-square angle of *σ*_D_ = 60°. We considered rotations around the *σ_xx_*, *σ_yy_*_,_ and *σ_zz_* principal axes, and around axes 30°, 40° and 50° apart from the *σ_zz_* principal axis. The results of these simulations are summarized in Figure 5. As observed, the spectral shapes change considerably with the rotation axis, which may allow such axes to be identified from the 2D Exchange Spectrum. 

**Figure 6:**
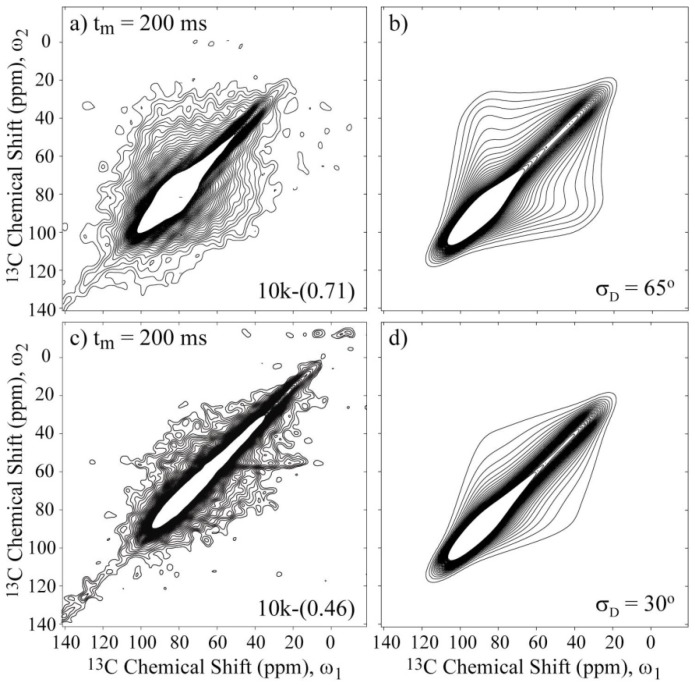
Experimental and simulated 2D Exchange spectra for the (**a**),(**b**) 10k-(0.71) sample at T = 20 °C and (**c**),(**d**) the 10k-(0.46) sample at T = 100 °C. The simulations were performed assuming rotations around the local chain axis with amplitudes distributed as Gaussian functions with rms values *σ*_D_ indicated in the figures. The orientation of the chemical shift tensor was assumed to be the same as that of crystalline PEG, and the conformational disorder was considered by calculating the reorientation angle distribution from the rotation angle distribution according to reference [36]. The temperatures were selected to ensure that the samples were in the same dynamic regime; see Figure 3a.

Figure 6a,c present the experimental 2D Exchange spectra for the 10k-(0.71) and 10k-(0.46) samples at 20 and 100 °C, respectively. When comparing the spectrum obtained for the 10k-(0.71) sample with the simulations presented in Figure 5, it can be observed that this spectrum resembles the majority of the spectra that were simulated with the rotation axis apart from *σ_zz_* axis with an angle on the order of 40° or above. The absence of well-defined elliptical ridges in the spectrum reveals that the motion does not occur between well-defined sites, which can be interpreted as a direct consequence of the conformational disorder along the polymer chains. Additionally, due to the spatial hindrance induced by the intercalation in the clay galleries, a two-dimensional dynamics is expected for the PEO chains. Furthermore, it was shown in reference [10] that PEO chains intercalated in clay assume an distorted helix conformation, exhibiting OC–CO gauche (~90%) and trans (10%) torsion angles. Thus, the motional model used was a rotation around the local helix axis. Considering that the *σ_zz_* principal axis is approximately equal to or greater than 40° displaced from the local chain axis, this result suggests that the molecular motions in the PEG intercalated in the clay is composed of rotations around the local chain axis of the distorted helix observed in reference [10]. In fact, because molecular reorientations transverse to the clay plates planes are unlikely due to the space restriction and planar wobbling of the chains would require the motion of many segments, these rotations around the local helix axis, which are similar to a helical jump but involve distorted sites, appear more favorable. As depicted in Figure 5, the spectra in Figure 5c,d are indistinguishable, showing that the uncertainty in the local axis orientation is about 20°. This is certainly a big uncertainty, but given the aforementioned features of the system reported previously, it seems reasonable to assume this geometry. Thus, the above identification of the rotational axis was performed by basically looking at the symmetry of the 2D Exchange spectrum. However, there are still some significant differences between the experimental and the simulated spectra. As previously mentioned, these differences might be attributed to the conformational disorder of the PEG chains, which was not considered in the simulations. 

However, it was demonstrated that for systems with considerable configurational and conformational disorder, executing uniaxial reorientations on the reorientation angle distribution,* R*(Δ*φ*), which fully determines the exchange intensity, is the autocorrelation function of the distribution of angular positions $p(\varphi -\bar{\varphi })$ in an ensemble of equivalent segments [36]. Thus, for a segment that has a probability *p*(*φ*_1_) of starting its motion at an orientation *φ*_1_ and whose corresponding reorientation angle distribution is *p*(Δ*φ* + *φ*_1_), *R*(Δ*φ*) is obtained as follows:

(1) $$R\left(∆\varphi \right)=\overset{2\pi }{\underset{0}{\int }}p\left(\varphi_{1}\right)p(∆\varphi +\varphi_{1})d\varphi_{1}$$

For a specific motional amplitude, *φ*_D_, $p(\varphi -\bar{\varphi })$ is a box function with equal probability between *φ*_D_ and *φ*_D, _which leads to a triangular *R*(Δ*φ*) [36]. Then, to consider the configurational and conformational disorder, a Gaussian shaped amplitude distribution *A*(*φ*_D_) is assumed and the final *R*(Δ*φ*) is obtained by the summation of the triangular *R*(Δ*φ*) function for each *φ*_D*.*_

Figure 6b presents the simulated spectrum obtained using this *R*(Δ*φ*) obtained for a motional amplitude of *σ*_D_ = 65° and a CSA tensor orientation of (90°, 30°, and 90°). As observed, the agreement between the experimental and simulated spectra is considerably improved; the conformation disorder is significant in this system, as previously suggested in reference [10]. Also note that a reorientation angle distribution given by Equation (1) has a high intensity at small angles that decays for higher angles. Thus, it can also be a good model for a system that has some segments that exhibit small angle motion and other segments that exhibit high angle reorientations. Notice that, as usual in Exchange NMR experiments, this result is also equivalent to motions that occur with a large distribution of correlation times, where segments that move outside or in the limits of the dynamic range detected by Exchange NMR (ms–s) contribute as if they were rigid or exhibit small angle motions [37,38]. In both interpretations, the models fit with the PEO-montmorillonite anchoring model proposed in references [18,19,20,21,22]. Specifically in tightly confined environments, it may be difficult for long-chain molecules to adopt their lowest energy conformation, and they expect to find low-amplitude reorientation enhanced due to inefficiencies in packing and lack of access to low energy conformations. However, these effects, which are expected to lead to enhanced dynamics, must be balanced by the interactions of the organic layer with the inorganic surface. The attractive interactions that stabilize the interface tend to pin the polymer to the surface at multiple sites along its backbone, and these pinned sites inhibit the large-angle reorientation of the polymer in these slit pores. Therefore, despite the differences between the PEG/Na^+^-hectorite and PEO/Li^+^-montmorillonite intercalation compounds, which includes both cation capacity exchange and the interlayer cations, we expect to observe similar anchoring effects for the PEG/Na^+^-hectorite nanocomposite. In the anchoring model, the cations on the surface of the clay serve as pinning points for the oxides in PEG, where multiple oxygen atoms try to associate with each such charge, and as a result of geometric constraints in the vicinity of the pinning, the layer of PEG closest to the surface is (i) distorted with respect to its local conformation compared to the bulk and (ii) dynamically inhibited compared to bulk. Furthermore, for those PEO/PEG segments which are not pinned to a surface, the awkwardness of packing in the vicinity of the confining wall leads to an effective density gradient and somewhat inefficient packing, which leads to increased dynamics, at least relative to the bulk models. 

Using the above model and procedure to perform the simulations, we investigated the influence of the chain lengths and packing (single- or double-layered) on the geometry of the slow molecular motions. Figure 6c,d present the experimental and simulated 2D Exchange spectra for the 10k-(0.46) sample. In this case, the amplitude of the motion is reduced to *σ*_D_ = 30°, which is considerably less than the value obtained for the 10k-(0.71) sample. This result indicates that the direct interaction of the polymer with both clay platelets has a significant influence on the PEG dynamics, which most likely restricts the motion of the chain near the surface that consequently increases the contribution of conformations associated with small angle motion in the reorientation angle distribution or the number of anchoring sites.

**Figure 7:**
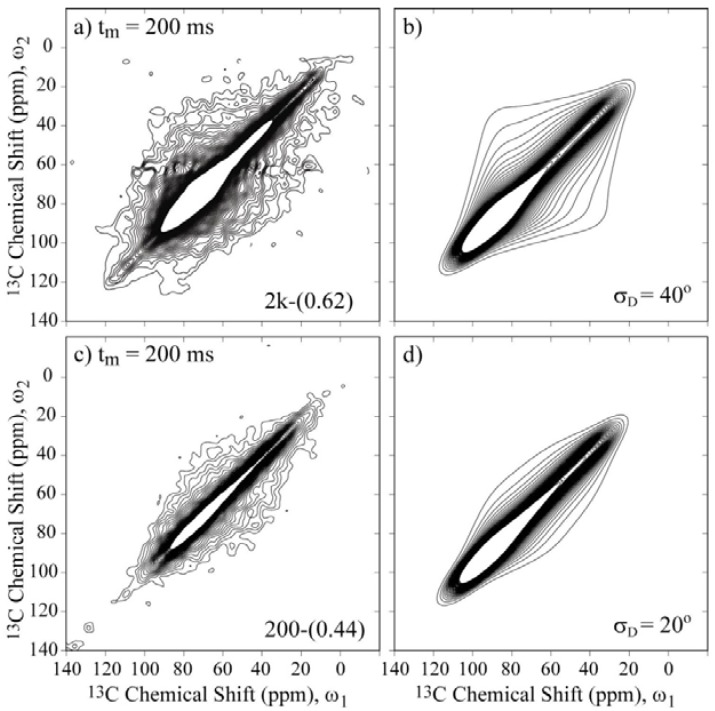
Experimental and simulated 2D Exchange spectra for the (**a**),(**b**) 2k-(0.62) sample at T = −20 °C and the (**c**),(**d**) 200-(0.44) sample at T = 10 °C. The simulations were performed assuming rotations around the local chain axis with amplitudes distributed as Gaussian functions with rms values *σ*_D_ indicated in the figures. The orientation of the chemical shift tensor was assumed to be the same as that of crystalline PEG, and the conformational disorder was considered by calculating the reorientation angle distribution from the rotation angle distribution according to reference [36]. The temperatures were selected to ensure that the samples were in the same dynamic regime.

Figure 7 presents the 2D Exchange spectra of the 200-(0.44) and 2k-(0.62) samples. These results allow the determination of how the chain length affects the motional amplitudes in the single- and double-layer configurations. By comparing Figure 6a with Figure 7a and Figure 6c with Figure 7c, it is evident that the overall amplitude of the motions increases with the molecular weight. Based on the anchoring model [18,19,20,21,22], this result indicates that the anchoring points are hindering the PEG motion because this effect is more evident for smaller molecular weights, where the restriction to the motion is more effective for extending the full polymer length, and for single-layer configurations, where an increase of anchoring points is expected due to the interaction of the polymer chain with both clay layers.

To confirm the model proposed above, we performed Centerband-Only Detection of Exchange (CODEX) [24,25] experiments and used the same model to fit the experimental data. This technique allows the slow molecular motions in a sideband-free 1D MAS spectrum to be identified and characterized. More specifically, the MAS spectrum measured after the application of the CODEX pulse sequence [24,25] depends on the difference of the CSA dependent MAS phases (recoupled by a REDOR like train of π pulses), which are encoded during two evolution periods with a duration of *Nt*_r_ that flanks a mixing period *t*_m_. To avoid intensity decay due to relaxation during the evolution and mixing periods and to remove the non-mobile part of the signal, the CODEX spectral intensities are usually subtracted and normalized by the intensities of a reference spectrum *S*_0_, which is acquired with the same pulse sequence but with a mixing time that is considerably shorter than the motion correlation times; therefore, no motion effects are codified in this spectrum. The result is a pure-exchange intensity Δ*S* = (*S*_0_−*S*)/*S*_0_, where the *t*_m_ dependence and the *Nt*_r _provide the motion correlation function and the motional amplitudes, respectively.

**Figure 8:**
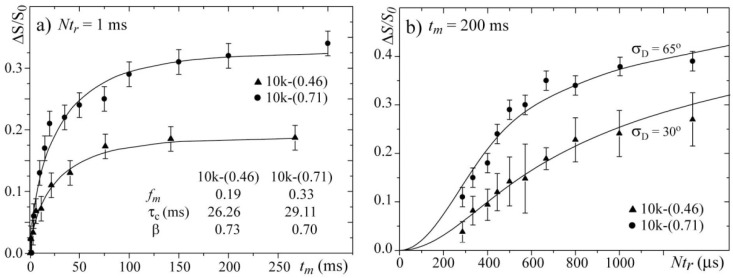
(**a**) Mixing time *t*_m_ and (**b**) evolution time *Nt*_r_-dependences of the CODEX normalized intensities for the 10k-(0.71) sample at T = 20 °C and the 10k-(0.46) sample at T = 100 °C.

Figure 8a presents the *t*_m_-dependences of the normalized pure-exchange CODEX intensities ∆S/S for the 10k-(0.46) and 10k-(0.71) samples. Both curves were fit using stretched exponential functions. Note that the parameter *τ*_c_ and the stretching parameters *β* obtained from the *t*_m_-dependence are very similar for both samples. This result confirms that by selecting the temperatures such that the line widths are motion narrowed to similar values, the dynamics of the chains in both samples occur with similar correlation times,* i.e.*, they are occurring in comparable motional regimes.

Figure 8b presents the ∆S/S *Nt*_r_-dependences for the 10k-(0.46) and 10k-(0.71) samples and the corresponding simulations. In the simulation, the same model and parameters used for simulating the 2D Exchange spectra shown in Figure 6 were used. For both samples, the agreements between the experimental and simulated curves are reasonably good. The differences between the *Nt*_r_ CODEX curves of both samples are clear, which confirms that the amplitude of the rotations is smaller for the sample with the single-layer intercalation. In fact, the results obtained by the CODEX might appear redundant because the 2D Exchange experiments provided the same information, but this is not totally true. 2D Exchange is a non-normalized experiment, and a decrease in the motion amplitude can be confused with a decrease in the fraction of segments detected in the time window probed by the experiment (mobile fraction). As an example, let us consider a system were the motion amplitude is sufficiently high to produce a 2D Exchange spectrum with an amplitude in all spectral ranges. Nevertheless, for diffusive types of molecular motion, the intensities in the wings of the 2D Exchange spectrum (spectral regions well off diagonal) are usually considerably less than in the near diagonal regions. Consequently, if only a small fraction of the molecular segments participate in the exchange process, even for high angle motions, the intensity in the wings in the 2D Exchange spectrum might be below the noise level, which may give the impression that the motion amplitude is smaller than it actually is. This behavior may bias our interpretation concerning the differences in the motional amplitudes because the mobile fraction is also considerably different among the samples. Here, the CODEX results play their major role. Because of the normalization, in the CODEX experiments, the information about the mobile fraction and the motional amplitudes are uncoupled,* i.e.*, while the plateau in the *Nt*_r_ dependence provides the *f*_m_, the slope of such curve depends on the rotation angle. Therefore, the clear difference in the slope of the curve shown in Figure 8b reveals that there is indeed a difference in the motional amplitudes of the two samples, which validates the analysis and interpretations of the 2D Exchange experiments presented above. Finally, we would like to point that, due to the aforementioned reasons, a precise evaluation of reorientation angle and distribution is difficult to be done, so the proposed motion geometry was based on both the experimental results and the unique characteristics of the system. However, most of the general conclusions obtained by comparing the different samples are indeed model free, being the simulations done to quantify the results and establish the most probable (in our view) picture for the motion in these systems. 

## 3. Experimental Section

### 3.1. Materials

Poly(ethylene glycol) (PEG) with a molecular weight of 10,000 g/mol was purchased from Aldrich, whereas those with molecular weights of 2000 and 200 g/mol were purchased from Fluka. 

The Na^+^-hectorite (SHCa-1) from the Source Clay Minerals Repository at the University of Missouri–Columbia, was prepared for the intercalation process using procedures similar to those described elsewhere [7,8,10,28]. Na^+^-Hectorite (HCT) was first treated with 0.5 N acetic acid to eliminate carbonate impurities. Afterwards, the clay was dissolved in deionized water and filtered several times [28]. No ion exchange was performed. After this purification process, the Na^+^-clays were dried in an oven at 65 °C and then heated to 80 °C under vacuum for several days. These dried Na^+^-clays, which were sealed in capillary tubes, were analyzed by WAXD to determine the interlayer gap. 

The nanocomposites were obtained from the stoichiometric addition of PEG and Na^+^-clays in acetonitrile. Two reaction stoichiometries were used for the preparation of the layered nanocomposites: R = 0.1 and 0.4 (R = g PEG/g clay) to obtain uniform gallery expansions of 0.4 and 0.8 nm, which were associated with the formation of single and double PEO layers, respectively [7]. These reactions involved 300 or 150 mg of clay and 30 or 60 mg of PEG dissolved in 20 mL of acetonitrile. The colloidal suspension was stirred for *ca*. 24 h at room temperature, filtered, and then the nanocomposite was dried following the procedure described above. These Na^+^-clay nanocomposites were immediately sealed in appropriate glass tubes for NMR and WAXD analyses and aluminum pans for DSC analysis. For the stoichiometry of R = 0.4, the excess PEG was eliminated by repeatedly washing with deionized water [28], and the DSC measurement did not reveal a melting peak.

The nomenclature used to identify the samples was M_w_ − (Δc), where M_w_ is given in g/mol and Δc = c_1_ − c_0 _is the interlayer gap, given in nanometers (nm), where c_0_ and c_1_ are the long periods of the inorganic layers before (host) and after intercalation (nanocomposite), respectively, as determined by WAXD (see Figure 1). Pure PEG with a molecular weight of 10,000 mol/g was denominated PEG-10k. Table 1 summarizes the series of samples prepared for this work.

Like most naturally occurring silicates, hectorite contains paramagnetic impurities, which induces line broadening in ^13^C NMR spectra and reduces the spin-lattice relaxation times [10,14].

### 3.2. Methods 

WAXD studies were performed using a Rigaku Rotaflex RU 200B instrument (Cu Kα radiation, *λ =* 1.5418 Å). DSC measurements were performed using 10 mg samples loaded in Al pans on a Model 2920 Modulated Differential Scanning Calorimeter from TA Instruments. The samples were heated at a rate of 10 °C/min from room temperature to 90 °C under a nitrogen atmosphere. 

The NMR experiments were performed using a VARIAN INOVA spectrometer at ^13^C and ^1^H frequencies of 100.5 and 400.0 MHz, respectively. Static experiments were performed using a double-resonance DOTY Science Instruments probe head with variable temperature (VT). CP/MAS experiments were performed using a VARIAN 7 mm MAS double-resonance probe head with VT. The spinning speed was controlled using a VARIAN pneumatic system that ensures a rotation stability of ±2 Hz. Typical π/2 pulses lengths of 3.5 and 4.0 µs were applied for ^13^C and ^1^H, respectively. A proton-decoupling field strength of 60 kHz, cross-polarization time of 1 ms and recycle delays of 1 s were used. The time scale and amplitude of the slow molecular motions were investigated using CODEX (Centerband-Only Detection of Exchange) [24,25] experiments with mixing times (*t*_m_) ranging from 1 to 700 ms and evolution times (*Nt*_r_) ranging from 333 to 2500 µs. 2D Exchange experiments [23] were conducted using a mixing time of 200 ms and acquired with 128 t_1_/ω_1_ points. The orientation of the CSA principal values was estimated using 2D Separated-Local-Field (SLF) experiments [26,27] acquired with 64 t_1_/ω_1_ points_._ For the SLF experiment performed for pure PEG, the signal of the amorphous phase was filtered by applying a *z*-filter with duration of 1 s immediately after the CP excitation. The temperature for all experiments was calibrated using Pb(NO_3_)_2_ according to the procedure described in reference [39]. 

## 4. Conclusions

In this study, we used Exchange NMR methods to study the motional amplitudes of polymers in intercalation PEG/Na^+^ Hectorite nanocomposites and compared the different polymer layer configurations and molecular weights.

The results revealed the presence of small and large angle reorientations of the PEG chains, which indicated the presence of segments with restricted dynamics that were most likely due to the anchoring points between the clays platelets and the PEG oxygen atoms induced by the Na^+^ cations.

For the single-layer configurations, the amplitude of the motions of the PEG chain was considerably less than that observed for the double-layer intercalation compound. This result indicates that the effect of having the polymer chain interacting with both clay platelets is stronger, which can be understood as the effect of increasing the number of anchoring points. 

While comparing samples with the same number of polymer layers, but with different molecular weights, it was observed that the overall amplitude of the motions increased with molecular weight. This result confirms that the anchoring points hinder the motion of the PEG, and this effect was more evident for the PEG with smaller molecular weights, where the restriction to the motion is more effective in extending the full polymer length. 
